# Predictors of Psychoactive and Nootropic Substance Use Among Romanian University Healthcare Students: The Interplay Between Academic Anxiety and Educational Advancement

**DOI:** 10.3390/healthcare14142079

**Published:** 2026-07-11

**Authors:** Ioana-Cezara Caba, Magdalena Iorga, Iustina-Gabriela Mihăianu, Luminița Agoroaei, Alexandra Jităreanu, Raluca Ortensia Cristina Iurcov

**Affiliations:** 1 Faculty of Pharmacy, Grigore T. Popa University of Medicine and Pharmacy, 700115 Iasi, Romania; ioana-cezara.caba@umfiasi.ro (I.-C.C.); luminita.agoroaei@umfiasi.ro (L.A.); jitareanu.alexandra@umfiasi.ro (A.J.); 2 Faculty of Medicine, Grigore T. Popa University of Medicine and Pharmacy, 700115 Iasi, Romania; nutr-rom-514@students.umfiasi.ro; 3 Faculty of Psychology and Educational Sciences, “Alexandru Ioan Cuza” University, 700554 Iasi, Romania; 4 Faculty of Medicine, University of Oradea, 410087 Oradea, Romania; riurcov@uoradea.ro

**Keywords:** cognitive enhancers, smart drugs, nootropics, university students in healthcare fields, stress and academic pressure, drug abuse

## Abstract

**Background:** The use of cognitive enhancers (CEs) is becoming increasingly widespread among students, especially in health fields, with significant implications for both academic performance and student well-being. The aim of the study was to investigate the use of CEs among healthcare university students in relationship with stimulant intake, academic progression, and academic anxiety. **Material and Methods:** The cross-sectional study included 402 students enrolled in medical studies (general medicine, pharmacy, dentistry, and physiotherapy). Socio-demographic characteristics, health-related and academic data, lifestyle behaviors, and the consumption of different CEs were collected. The Academic Anxiety Scale was used to evaluate university students’ perceived stressors that contribute to academic anxiety. Data processing analysis was performed using IBM Statistical Package for Social Sciences. **Results:** The frequency of cognitive compound intake exhibits a positive correlation with the consumption of group B vitamins (r = 0.170, *p* = 0.001), tea (r = 0.158, *p* = 0.003), and coffee (r = 0.113, *p* = 0.031). *Lecithin* and *Ginkgo biloba* were significant independent predictors (*p* < 0.05) of enhanced alertness, memory, and academic performance. The only significant independent predictor of cardiovascular events (palpitations, tachycardia) is caffeine consumption, which increases the risk by nearly three times (OR = 2.96; *p* = 0.001). In terms of addictive risk, drinking caffeine raises the likelihood of being addicted by 4.78 times (*p* = 0.014). It has been demonstrated that the probability of using synthetic nootropics (piracetam/cerebrolysin) increases by 1.86 times (OR = 1.86, *p* = 0.021) as university education progresses (nN= 335). Academic anxiety had a mean score of 23.27 ± 7.38. Unemployed students exhibit markedly elevated anxiety levels compared to employed students. Respondents who justify the use of stimulants by feeling stressed or overwhelmed present the highest levels of academic distress, in contrast to those who use them to combat fatigue or out of curiosity. A large majority (95.0%) do not consider use of CEs as a form of cheating on examinations, and 88.9% are against its prohibition. **Conclusions:** The findings of this study highlight that many university students in the healthcare field use cognitive stimulants during periods of intellectual overload, with more than 40% of participants utilizing CEs daily or weekly in periods of academic stress. Overload schedule, academic stress, the knowledge about their positive effects, and psychosocial contexts influence consumption.

## 1. Introduction

The term “cognitive enhancers” covers a broad spectrum of substances used to optimize mental performance. They vary drastically in terms of pharmacology and safety. The most commonly used by the general population are dietary stimulants (such as coffee, tea), nutritional supports (such as vitamin supplements, frequently consumed for transient alertness or metabolic support with minimal physical risk and little or no potential for addiction), and phototherapeutic agents, such as herbal products consumed for zero risk of addiction, but potential drug interactions are often ignored. Higher levels of synthetic nootropics and amphetamines are also used as cognitive enhancers (CEs). The former improves memory and learning and supports long-term brain health with a cumulative effect and without a sudden state and carries a low to moderate risk of addiction, while the latter cause severe cardiovascular overload and physical dependence. Administration of pharmacologically active stimulants can be associated with cardiovascular effects (tachycardia, hypertension), neuropsychiatric symptoms (anxiety, insomnia), and potential dependence [1,2,3,4,5].

The use of cognitive stimulants, also known as “smart drugs,” “study aids,” or “nootropics,” among students is widespread, motivated by academic performance and peer influence but raises significant health and ethical concerns. In highly competitive academic environments, the use of cognitive enhancers (CEs) is intense or even abusive with the intention of obtaining important academic results. Concerns arise about their inappropriate use and the associated health risks [1,2]. The use of cognitive enhancers includes both prescription drugs (methylphenidate, modafinil, synthetic nootropics) and over-the-counter substances (herbal extracts, neuroprotective agents, vitamins and essential minerals, amino acids and metabolic precursors, etc.). Depending on demographic factors, including gender, year of university study, and field of study, males report higher rates of use [1,2,3,4].

Students primarily use cognitive enhancers (CEs) to improve their concentration, alertness, and academic performance due to factors such as high academic pressure and competition. Social influences, such as peer use, play a significant role in motivating students to use these substances. Also, many students believe that CEs can improve study efficiency and exam performance, perceiving CEs as a necessary tool for success [2,3,4,5].

The misuse of cognitive enhancers by students poses significant health problems: the potential for addiction, adverse side effects, and long-term health implications [2,3,4,5].

The prevalence and types of cognitive enhancers used vary significantly across geographic regions. In the Middle East, the use of cognitive enhancers is moderately high, influenced by academic pressure, with easy accessibility, with or without a medical prescription. Their use is also frequently associated with a strong perception that they improve academic performance [4,6], but they are also used to deal with anxiety.

Anxiety is a normal emotion, is future-oriented, and is a common stress response. Anxiety is characterized by emotional, mental, and physical symptoms, such as fear, difficulty concentrating, or increased heart rate. Anxiety is considered excessive or pathological when it is excessive or prolonged and when the negative impact on psychological, social, occupational, and biological well-being is significant [7].

Academic anxiety is a specific domain of anxiety that is determined by the academic context and is experienced by learners in educational settings [8]. Academic anxiety has been identified as a preclinical indicator of anxiety that provides important predictive utility to clinical symptomologies such as anxiety and depression.

High rates of academic anxiety have been registered among students. In 2022, the American College Health Association identified that half of the students (63.40 %) experienced overwhelming anxiety at some point in their last year [9]. The prevalence of anxiety among European, American, and Asia-Pacific college and university students is relatively high, with scores between 31% and 50% [10]. Several studies conducted during and after the COVID-19 pandemic identified even higher rates, showing that more than 80% of students presented high scores for stress and anxiety [11,12,13].

In a meta-analysis published by Mao et al., the research team identified a prevalence of general anxiety that ranged from 8.54% to 88.30% among medical students. The scores were identified as coming from articles published between 2000 and 2018 [14].

Many other researchers in the field have identified a strong relationship between academic anxiety and physical symptoms (increased heart rate), lifestyle, and psychological and behavioral changes such as absenteeism, low self-esteem, depression or chronic stress, sleep-related problems, consumption of alcohol, caffeine, supplements, or illicit drugs [13,15,16]. The higher rates of academic anxiety are strongly linked to lack of concentration, difficulties in learning engagement, and poor academic results.

A large scientific literature shows that anxiety symptoms are significantly more prevalent among medical students than non-medical students or the general population [17,18,19,20]. In Western Europe (e.g. Portugal, the Netherlands, the United Kingdom), moderate use of CEs is reported; over-the-counter supplements and “soft enhancement” strategies are more commonly used, and strict drug regulation and high awareness of risks are described [21,22,23]. In New Zealand, the prevalence of use is relatively stable, with permissive attitudes towards use in the university environment, a competitive academic culture, and, at the same time, a reduced perception of risk [24,25]. Substance categories vary significantly across regions. In European countries and Switzerland, combinations of prescription drugs (e.g., methylphenidate), recreational drugs, and “soft enhancers” (caffeine, supplements) are used [26]. In Northern and Eastern Europe (e.g. Lithuania), the prevalence of use of medicinal compounds is lower, suggesting either more limited access or a lower social tendency to accept these compounds [5]. However, underreporting and the use of non-prescription substances may mask chronic exposure at low doses with negative health impacts.

In contrast, Western European countries (e.g., Portugal, the Netherlands, the United Kingdom) show a trend towards mixed-use patterns, combining pharmacological agents with “soft enhancers” such as caffeine supplements or nootropic compounds. Even if perceived as safe, they can raise issues of toxicity. Natural supplements and mild enhancers (caffeine, herbal nootropics) induce toxicity when used chronically or in high doses. For example, high caffeine consumption can induce sleep disorders, increased cortisol levels, cardiovascular stress, and decreased cognitive performance [21,22].

For the general population, the distinct classes are only interested in achieving the desired effects. But for health students, the differences are analyzed in terms of benefits and risks, due to their professional knowledge. Students represent a vulnerable category of abusive users of ECs, due to their desire to achieve performance, with a prevalence rate between 5.3% and 35% [1,2,3,4,5]. The percentage of medical students identified in various studies is up to 47.4%. High levels of academic stress, pressure to perform, and the need to get high grades are among the most common reasons for abusive use [27,28].

In the context of polydrug use, Hawas et al. describe the increased risks of misuse, cumulative exposure, and drug interactions [1]. In some studies, an association between stimulant use and eating disorders or mental health has been described, suggesting a more complex risk profile than simple cognitive optimization [29].

Regarding ethical and risk perceptions, there are notable differences between different regions. In Europe, there is a tension between performance and ethics, especially among health students [30]. In Australia and New Zealand, students recognize the risks but consider using CEs in certain academic contexts. In some developing countries, perceived risk is lower, and use is more often considered acceptable for academic success, reflecting cultural and educational differences [25,27]. From an ethical perspective, using CEs for academic advantage raises questions about whether such practices constitute fraud [31]. There are concerns about increased awareness of the risks associated with CE use and educational initiatives to promote healthier study habits among students [1,2,4,5].

The use of cognitive enhancers among students is widespread, explained by academic performance support and peer influence, but raises significant health and ethical concerns. Addressing these issues, it is crucial to highlight the risks associated with continued use. Despite the growing number of international studies, there remains a significant data gap in Eastern Europe. Research about the use of cognitive enhancers among healthcare students in Romania is scarce. A cross-sectional study developed in 2021 aimed to determine the use of neuroenhancers and the motivations and factors associated with their use in French and Romanian university students. The results showed the prevalence of drugs of abuse consumption to be about 8% among Romanians students and 3% among French students [29].

The aim of the present study was to identify how the use of CEs correlates with self-assessed cognitive performance, cardiovascular and addiction risks, and academic anxiety and students’ ethical perception of substance use. The hypotheses of the study are:

**H1.**  *Academic anxiety will exhibit a significant positive association with the probability of use of amphetamines or other psychoactive substances.*

**H2.**  *Academic advancement will exhibit a significant negative association with the use of amphetamines or other psychoactive substances.*

**H3.**  *Academic advancement will exhibit a significant positive association with the use of synthetic nootropics such as piracetam and cerebrolysin.*

**H4.**  *Academic anxiety will not exhibit a significant positive association with the use of synthetic nootropics.*

## 2. Materials and Methods

### 2.1. Participants and Study Design

A cross-sectional study was conducted between December 2025 and January 2026, among students registered at “Grigore T. Popa” University of Medicine and Pharmacy in Iași (UMF Iași) and Faculty of Medicine, University of Oradea, Romania.

The questionnaire was constructed specifically for the present study. The questionnaires were distributed online to students enrolled in the healthcare field (general medicine, dentistry, pharmacy, nursing, physiotherapy, and nutrition and dietetics, all years of study, all ages and genders. The online questionnaires, specially constructed for this study, included constructed items and a psychological instrument (Academic Anxiety Scale) and were distributed to the students responsible for the study year. Completing the questionnaire was voluntary, anonymous, and implied consent to participate in the study. Students were informed about the purpose of the study and the confidentiality of the data and that they could withdraw from the study at any time without consequences. Respondents were not offered incentives. Informed consent was included at the beginning of the questionnaire. The inclusion criteria required the status of active students. The exclusion criteria targeted forms sent after the deadline. Following the application of these criteria, the final sample included 402 valid respondents.

### 2.2. Research Tool

Data were collected through an online questionnaire, developed using the Google Forms platform. The instrument was structured in three main sections, as follows:

- The first section recorded socio-demographic and academic variables, such as gender, age, background, professional and marital status, year, and program of study. Self-reported anthropometric parameters, namely weight and height, were collected to calculate the body mass index (BMI). More items were constructed to collect information about lifestyle behaviors were assessed by quantifying the frequency of tobacco, alcohol, coffee, energy drink, and drug consumption, as well as the average number of hours of sleep per night and the presence of chronic conditions (answers yes/no or 1 (never to 4 (daily)).
- The second section measured the level of stress and academic pressure using the Academic Anxiety Scale [17]. This included 11 items targeting aspects such as performance concerns, fear of failure, or procrastination, rated on a 4-point Likert scale, ranging from 1 (“Not at all typical of me”) to 4 (“Very typical of me”). This scale constructed by Moreire de Sousa et al. is effective for evaluating university students’ perceived stressors that contribute to academic anxiety. The total scores represent low/not anxious (11–21), moderate anxiety (22–32), and high academic anxiety (33–44). Following the analysis, Cronbach’s alpha was 0.888 for the 11 items, indicating very good internal consistency for the assessment tool.
- The third section assessed the consumption of psychoactive substances and cognitive stimulants, using items developed by the authors based on a review of literature regarding student cognitive enhancement behaviors. Data were collected on the origin of the administered compounds, explicitly differentiating between natural or over-the-counter options (such as *Ginkgo biloba*, guarana, coffee, and vitamin B complex) and synthetic or prescription-based medicinal substances (such as piracetam, cerebrolysin, and amphetamines). Subsequent items with yes/no answers, especially constructed for this study, assessed the reasons for administration, such as stress, curiosity or overexertion; the perceived beneficial effects, such as increased concentration and short- or long-term memory; and the presence of adverse effects, including cardiovascular disorders, insomnia, tolerance, or addiction. These specific variables, were designed to capture usage frequencies, procurement channels, and the students’ ethical perceptions regarding the use of these compounds as a form of academic fraud.

### 2.3. Statistical Analysis

Data processing analysis was performed using IBM Statistical Package for the Social Sciences (SPSS) for Windows, version 26 (SPSS Inc., Chicago, IL, USA). Continuous variables were described by means (M) and standard deviations (SD), and categorical variables by absolute and relative frequencies (percentages).

Sample sizes fluctuated across specific analyses due to incomplete or optional responses. A pairwise deletion approach was implemented to handle missing observations and preserve maximum statistical power.

Predictors for the binary and multiple logistic regression models were selected based on theoretical relevance and preliminary significant bivariate associations (*p* < 0.05). Categorical variables were dummy-coded (0 = absence/reference group, 1 = presence). Multicollinearity was assessed using Variance Inflation Factors (VIF), with all values remaining below 2.0, indicating the absence of significant collinearity. Model goodness-of-fit and performance were evaluated using the Omnibus test of model coefficients (*p* < 0.05) and the Nagelkerke pseudo-R^2^ statistic. Specifically, binary logistic regression models were executed to predict the probability of adverse effects and perceived cognitive benefits. Furthermore, to evaluate the structural pathways linking academic anxiety to sleep deprivation, a mediation analysis was conducted using Model 4 of Hayes’ PROCESS macro (v4.0) with 5000 bootstrap resamples to compute 95% confidence intervals.

Additionally, multiple logistic regression analyses were conducted to determine the simultaneous predictive value of specific adaptogenic substances (*Ginkgo biloba* and *Lecithin*) on self-reported cognitive domains, including perceived attention/vigilance, short-term memory, long-term memory, and enhanced academic performance. For each model, unstandardized regression coefficients (B), standard errors (SE), Wald statistics, and odds ratios (OR) with their corresponding 95% confidence intervals (CI) were calculated.

Body mass index (BMI) was calculated for each participant and used as an indicator of weight status classification, in accordance with the World Health Organisation recommendations, adapted to the European population. Thus, BMI values were classified into the following categories: underweight for BMI < 18.5 kg/m^2^, normal weight for the range 18.5–24.9 kg/m^2^, overweight for 25.0–29.9 kg/m^2^, class I obesity for 30–34.9 kg/m^2^, class II obesity for 35.0–39.9 kg/m^2^ and class III obesity for values ≥ 40 kg/m^2^ [32].

Data normality was assessed using the Kolmogorov–Smirnov test, which revealed that continuous and ordinal variables—specifically age, body mass index (BMI), global academic anxiety scores, and substance consumption frequencies—did not follow a normal distribution (*p* < 0.05). Consequently, non-parametric statistics were applied, and homogeneity of variances was evaluated using Levene’s test prior to conducting comparative analyses. The Mann–Whitney U test was used to compare independent groups based on gender (male vs. female), professional status (employed vs. unemployed), and non-prescription stimulant use (users vs. non-users). Comparative analysis for variables with more than two categories was performed using the Kruskal–Wallis H test. To determine the relationships between ordinal and continuous variables, the Spearman correlation coefficient (r) was calculated. The statistical significance level was set at *p* < 0.05. Chi-square (χ^2^) tests of independence were applied to evaluate the associations between categorical variables, specifically analyzing the impact of information sources on non-prescription procurement methods, as well as the relationships between sleep patterns, energy drink consumption, and stimulant-induced insomnia. Throughout the text, the numbers reported within parentheses for both H and χ^2^ tests represent the corresponding degrees of freedom (*df*).

The internal consistency of the 11-item Academic Anxiety Scale was evaluated using Cronbach’s alpha coefficient.

### 2.4. Ethical Approval

The research adhered to the ethical principles set out in the Declaration of Helsinki. The study was approved by the Research Ethics Committee of the University of Medicine and Pharmacy, Oradea, Romania (No. 21129/22 December 2025).

## 3. Results

### 3.1. Socio-Demographic, Anthropometric, and Lifestyle Data

The study included 402 medical students, with a mean age of M = 21.98, SD = 2.11 years. The distribution by year of study is relatively uniform, with respondents enrolled in the first to fifth years (with a maximum weight of 24.6% in the fifth year). Regarding the duration of the university programs they follow, half of the students (50.0%) are enrolled in 6-year programs (General Medicine, Dentistry), 37.6% are enrolled in 5-year programs (Pharmacy), and the remaining 11.9% are enrolled in 3- or 4-year programs. The detailed characteristics of the sample, including demographics, background, and an assessment of general health, are presented in Table 1.

The Mann–Whitney U test revealed significantly higher BMI values among male students (Mdn = 24.73) compared to female students (Mdn = 21.78, U = 8789.00, z = −4.92, *p* < 0.001).

### 3.2. Usual Substance Use and Cognitive Stimulant Use

To outline the behavioral profile of students during periods of nervous overload, the consumption frequencies for a series of substances with psychoactive or stimulant potential were evaluated. Details are presented in Table 2.

Detailed analysis of cognitive stimulant use shows regular use among participants: over 40% use them weekly or daily. Students who sleep less than 6 h/night reported a higher incidence of stimulant-induced insomnia (32.1%) compared to those who sleep more than 6 h (20.9%) (χ^2^ = 5.25, *p* = 0.022).

For energy drink consumption, a positive, dose-dependent association was observed (χ^2^(3) = 19.39, *p* < 0.001) with the incidence of insomnia, increasing progressively from 18.1% in non-consumers to 80.0% in daily consumers. A similar trend was identified regarding coffee consumption (r = 0.120, *p* = 0.023). The use of compounds (piracetam, cerebrolysin) doubles the risk of insomnia (χ^2^(2) = 13.10, *p* = 0.001). The detailed outputs of these statistical analyses are synthesized in Table 3, where the numbers enclosed in parentheses denote the respective degrees of freedom (*df*) for each chi-square test.

The central motivation for consumption is the optimization of executive functions: increased concentration (44.9%) and improved memory (26.2%). Respondents confirmed the expectations: 75.7% report clear benefits, such as increased alertness (71.3%) and easier short-term information retention (65.1%). Although the proportion of those who directly associate stimulants with adverse effects (tachycardia, insomnia) is 13.9%, the percentage of students who experienced severe insomnia and anxiety during the period of exams is much higher (62.4%).

The frequency of cognitive compound administration (n = 362) is positively associated with the consumption of B vitamins (r = 0.170, *p* = 0.001), tea (r = 0.158, *p* = 0.003), and coffee (r = 0.113, *p* = 0.031).

From an ethical perspective, the use of stimulants is normalized among respondents: 95.0% do not consider their administration to be a form of cheating on exams, and 88.9% oppose their prohibition in universities. Detailed data regarding the specific usage patterns, procurement channels, and ethical perceptions of both natural and synthetic cognitive stimulants are presented in Table 4.

The impact of the source of information on non-prescription use was assessed using chi-square tests. Social group is a significant influencing factor (χ^2^ = 8.79, *p* = 0.003). A total of 75.6% of students informed by their close friends choose non-prescription use. A positive association was also observed for recommendations from medical staff (χ^2^ = 7.20, *p* = 0.007), with 71.2% of them purchasing stimulants without a prescription. The online environment showed no statistically significant association (χ^2^ = 0.63, *p* = 0.425).

### 3.3. Academic Anxiety Scale

**Figure 1:**
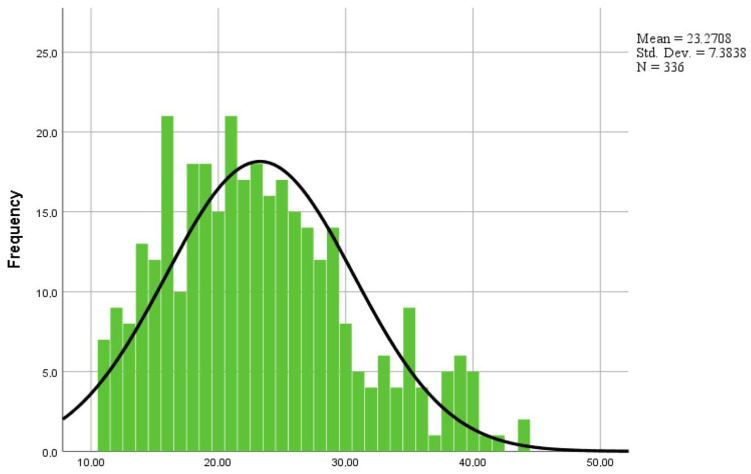
Distribution of the global academic anxiety score at the sample level (N = 336, M = 23.27 ± 7.38). The continuous black curve represents the theoretical normal distribution curve.

To assess students’ level of academic stress and pressure, the Academic Anxiety Scale was used. At the sample level (N = 336), the global academic anxiety score had a mean of M = 23.27, SD = 7.38, showing a moderate academic anxiety level. The distribution of results spanned the entire measurement spectrum, from a minimum of 11 points to a maximum of 44 points (see Figure 1).

The analysis indicated a distinct vulnerability by gender regarding academic stress. Female students recorded a significantly higher academic anxiety score (Mdn = 23.00) compared to male students (Mdn = 20.00, U = 7433.50, z = −2.95, *p* = 0.003).

The results obtained are directly associated with consumption behaviors. Students who resort to the use of non-prescription stimulants to support intellectual function present with significantly higher anxiety scores (Mdn = 23.00) compared to those who do not resort to such substances (Mdn = 21.00, U = 9907.00, z = −2.27, *p* = 0.023).

At the same time, students who fear developing an addiction have higher academic anxiety (Mdn = 26.00) compared to those who do not express such fears (Mdn = 21.00, U = 2863.50, z = −3.46, *p* = 0.001).

The presence of a chronic disease was also associated with a significantly higher level of anxiety (Mdn = 25.00, U = 5994.00, z = −1.99, *p* = 0.046) compared to the absence of such a pathology (Mdn = 22.00).

To assess variation in academic distress by the main reason for using cognitive stimulants, the Kruskal–Wallis test was applied. The analysis revealed a statistically significant difference, H(3) = 18.00, *p* < 0.001. Students who justify the use of stimulants by feeling stressed (Mdn = 24.00) or overwhelmed (Mdn = 23.00) present the highest levels of academic distress, in contrast to those who consume them to combat fatigue (Mdn = 21.00) or out of curiosity (Mdn = 17.00).

Unemployed students have significantly higher anxiety scores (Mdn = 23.00) than employed students (Mdn = 19.50, U = 4568.00, z = −3.43, *p* = 0.001). At the same time, students who live alone or in shared housing have higher academic anxiety (Mdn = 25.00 and 23.00, respectively) than those who live with their family (Mdn = 21.00, H(2) = 16.95, *p* < 0.001).

The assessment of the dynamics of academic distress during the academic years indicated a slight upward trend towards the final years (maximum level recorded in the fourth year). Still, the inferential analysis demonstrated that these variations were not statistically significant (χ^2^ (4) = 6.88, *p* = 0.142), with the level of anxiety remaining relatively constant.

The data analysis indicated a pattern of cumulative consumption used as a coping mechanism. The level of academic anxiety was positively and significantly correlated with the frequency of energy drink consumption (r = 0.113, *p* = 0.038) and carbonated beverages (r = 0.135, *p* = 0.014).

### 3.4. Regression Analysis

**Figure 2:**
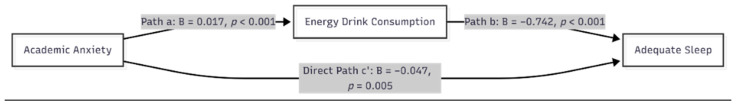
Mediation model illustrates the indirect effect of academic anxiety on sleep adequacy through energy drink consumption.

To further explore the mechanisms by which academic stress affects sleep, a mediation model (PROCESS macro v.4) was tested. Anxiety level positively predicts energy drink consumption (B = 0.017, *p* = 0.0001), which negatively predicts the likelihood of adequate sleep (B = −0.742, *p* = 0.0001). The direct effect of anxiety on sleep deprivation remained significant (B = −0.047, *p* = 0.005). A significant indirect effect was confirmed (effect = −0.013, 95% CI [−0.027, −0.003]), demonstrating partial mediation: anxiety instigates a stimulating behavior that exacerbates sleep deprivation. The structural pathways, unstandardized regression coefficients (B), and significance levels of this mediation framework are visually represented in Figure 2.

However, given the cross-sectional architecture of the study, these directional pathways must be interpreted with caution. This mediation framework represents a statistically legitimate exploratory association rather than an absolute causal mechanism, as temporal ordering among the variables cannot be definitively established.

A set of binary logistic regression models was developed to assess predictors for the occurrence of adverse effects. The predictive model was statistically significant (χ^2^ (4) = 23.402, *p* < 0.001), explaining 14.7% of the variance (Nagelkerke R^2^ = 0.147). Non-prescription self-medication practices significantly increase the risk of adverse outcomes by 3.05 times (OR = 3.053, 95%CI [1.081, 8.626], *p* = 0.035), whereas prescription-based procurement multiplies this likelihood by 5.12 times (OR = 5.125, 95% CI [1.881, 13.964], *p* = 0.001). Psychological concern regarding potential addiction also served as a significant independent risk factor, doubling the probability of reporting adverse events (OR = 2.296, 95% CI [1.403, 3.758], *p* = 0.001). Global academic anxiety scores did not reach statistical significance within this specific multivariate arrangement (*p* = 0.941). More details are presented in Table 5.

Regarding perceived cognitive efficacy, multiple logistic regression testing verified that *Ginkgo biloba* and *lecithin* stand as robust independent predictors across the analyzed sub-domains (*p* < 0.05). It is critical to note that the outcomes presented reflect the respondents’ subjective, self-reported evaluations regarding their cognitive capabilities (perceived benefits), rather than objective neuropsychological metrics of cognitive performance. However, the frequency of consumption of these plant extracts did not predict the global academic anxiety score (F(9, 326) = 1.51, *p* = 0.144), showing that none of the compounds with assumed adaptogenic action significantly modified the baseline anxiety levels. More details are presented in Table 6.

Caffeine consumption was identified as the only significant independent predictor for cardiovascular incidents such as tachycardia and palpitations, increasing the risk nearly threefold (OR = 2.958, 95%CI [1.594, 5.649], *p* = 0.001). Caffeine was also reconfirmed as a direct risk factor for sleep disturbances, multiplying the likelihood of insomnia (OR = 1.958, 95 % CI [1.218, 3.361], *p* = 0.015). Regarding substance dependence, caffeine intake raised the probability of addiction by 4.78 times (OR = 4.782, *p* = 0.014). Crucially, theanine utilization emerged as a powerful statistical predictor for both the development of pharmacological tolerance (OR = 4.965, 95 % CI [1.464, 16.843], *p* = 0.010) and psychological dependence (OR = 13.367, 95 % CI [3.541, 50.461], *p* < 0.001). More details are presented in Table 7.

The predictive model for the use of amphetamines or other psychoactive substances (n = 336) was highly statistically significant (χ^2^ (4) = 22.398, *p* < 0.001), explaining 32.0% of the variance in consumption (Nagelkerke R^2^ = 0.320). In behavioral and social sciences, an effect size explaining nearly one-third of the total variance is considered robust and reflects a highly meaningful predictive power for the combined academic and psychological factors. An increase in the academic anxiety score is associated with a higher probability of use (OR = 1.16, *p* = 0.007). In contrast, advancing in the year of study acts as a protective factor (OR = 0.42, *p* = 0.012). Gender (*p* = 0.113) and average hours of sleep (*p* = 0.276) did not contribute significantly. The specific parameters of this regression model can be observed in Table 8.

Regarding the use of synthetic nootropics (piracetam/cerebrolysin) (n = 335), the only significant independent predictor was year of study, with advancement in university education increasing the odds of use by 1.86 times (OR = 1.86, *p* = 0.021). Academic anxiety scores (*p* = 0.441), gender (*p* = 0.224), and hours of sleep (*p* = 0.078) did not reach significance.

## 4. Discussion

The use of cognitive enhancers (CEs) among students, particularly in the health sciences, is widespread. As described in the literature, the prevalence of use ranges from 50% in Europe to 70% and over in the Middle East and Africa. The differences can be explained by different perceptions across regions (including coffee and dietary supplements or not), study methodologies, and specific sociocultural factors [2,33,34].

In line with studies in the literature on European cohorts, our study highlights a weekly or daily use rate of over 40% among the 362 students who use them, among 402 respondents. In our study, cognitive enhancers were obtained mainly by friends and medical professionals. Also, our data analysis showed a pattern of cumulative consumption as a coping strategy. Other studies described how cognitive enhancers are frequently obtained through informal networks (friends, family) or via the internet, which lowers barriers to access and promotes unregulated use and insufficient awareness of the risks. Availability and easy access are major factors contributing to their use [3,35].

From a professional perspective, the use of these substances among future medical and pharmacy professionals is concerning, as they may themselves resort to off-label use, given that they will be the specialists responsible for prescribing and monitoring treatments [6,36].

Most studies report a frequent use of so-called “mild enhancers,” with caffeine-containing products being the most used substances. In comparison, pharmacological cognitive enhancers are used less frequently but strategically targeted during periods of peak cognitive demand [21,36]. This trend is also evident among students in Poland and Portugal, where coffee and energy drinks are the primary methods of cognitive enhancement [21,34,37]. Furthermore, consumption varies, ranging from occasional to regular use. 

In contrast, the use of prescription medications is lower but remains significant, particularly in highly competitive academic settings such as medical schools. In our study, the use of prescription stimulants accounts for up to 20% of positive respondents; this trend is also described in some studies, where the prevalence of prescription drug use for this purpose ranges between 5% and 20% [6,21].

Regarding the reasons for the use, students frequently report the need to improve attention, concentration, alertness, and academic performance, particularly during exam periods or times of intense stress [3,33,38]. In our study, the primary motivation for administration of these compounds was to optimize executive functions: increased concentration (44.9%) and improved memory (26.2%). These findings are correlated with those described by Pighi et al. in a study with Italian medical students, the use being oriented toward academic performance, increasing attention capacity, or combating fatigue states [38]. In our study, these expectations were confirmed by respondents: 75.7% reported clear benefits, such as increased alertness (71.3%) and easier retention of short-term information (65.1%). These results are supported by data from the literature describing a strong correlation between the use of CEs and academic pressure, as well as a performance-oriented culture [28]. Some studies described how students take these compounds to cope with deadlines and increased academic demands, which underscores the pressure of a competitive educational environment [21,34].

Analyzing the dynamics of academic anxiety during the university years, we found a slight upward trend towards the final years, but the inferential analysis demonstrated that these variations were not statistically significant, the level of anxiety remaining relatively constant. Our results are different from those identified by the research conducted by Cassady et al. [8] and Moreira de Sousa et al. [17].

The analysis of data showed that female students had higher rates of academic anxiety than male students. Similar findings were identified among medical students in different countries: Pakistan [39], Brazil [40], Saudi Arabia [41], and Portugal [17]. Surprisingly, purchasing prescription drugs increases the risk of adverse effects much more than self-medication, while a user’s psychological concern about addiction almost doubles the likelihood of reporting these events. These results could be due to a possible severe diagnosis among students who consult a doctor or a minimization of the consequences in the case of self-administration. Almost 3/4 (71.25%) of participants declared that they purchased stimulants without a prescription.

We identified that students who resort to the use of non-prescription stimulants to support intellectual function present with significantly higher anxiety scores. These results prove that students who are overwhelmed by academic activity and have higher rates of academic anxiety are more prone to using over-the-counter pills. Similar studies have shown that medical students use self-administered substances to improve their cognitive performance during exam sessions. Due to the large number of duties and academic pressure for high grades, medical and pharmacy students experience smart-drug consumption more often, poor sleep, and high levels of academic stress and anxiety. Poor sleep quality was correlated with high levels of anxiety and the use of prescribed drugs to cope with stress in the study of Alrashed et al. [42], conducted on medical students.

Users often underestimate the safety profile of cognitive enhancers. Although these substances are perceived as safe or beneficial, there is evidence of the potential for addiction, cardiovascular effects, sleep disorders, and neuropsychiatric complications [3,34]. In particular, a significant percentage of users report adverse reactions, such as tachycardia and insomnia, which contradicts the general perception of safety [34]. These findings are further supported by our study, in which, although the proportion of those who directly associate stimulants with adverse effects (tachycardia, insomnia) is 13.9%, the percentage of students who experienced severe insomnia and anxiety during the exam period is much higher (62.4%). The results of the present study showed that caffeine consumption increases the probability of addiction, and theanine is a significant predictor for the onset of addiction and the development of pharmacological tolerance. The large odds ratio observed for theanine and dependence (OR = 13.367) is due to an asymmetric distribution, where a small but highly concentrated sub-cohort of intensive users reported concurrent reliance during peak academic stress. This estimate serves as an exploratory risk indicator rather than definitive clinical proof of physical addiction.

It has been reported that caffeine improves attention and alertness in both adults and adolescents and that higher doses may enhance reaction speed [43,44]. Preclinical evidence exists about the impact of frequent caffeine intake on dopamine transmission. Caffeine interacts with dopaminergic pathways and may exacerbate addiction [45]. Additionally, the simultaneous administration of caffeine and theanine has been reported to enhance cognitive function and alertness, particularly attention, with no side effects [46].

A very important aspect, also, is the effect of cognitive enhancer use on sleep disorders, particularly insomnia. In our study, students who sleep less than 6 h per night report a higher incidence of stimulant-induced insomnia (32.1%) compared to those who sleep more than 6 h (20.9%) (χ^2^ = 5.25, *p* = 0.022). The percentage of students who experienced severe insomnia and anxiety during the exam period is relatively high (62.4%), even though only about 13% associate this with a possible adverse effect of cognitive enhancers. Interestingly, the use of piracetam and cerebrolysin doubled the risk of insomnia. These issues are also frequently reported in the literature: numerous substances for improving cognitive performance, particularly central nervous system stimulants, increase alertness and affect sleep quality [47,48]. Studies show that over 60% of users experience adverse effects, with sleep disturbances present in 40% of them [34,49].

Thus, the use of these substances can negatively affect sleep quality. In our study, coffee consumption was associated with the incidence of insomnia in a dose-dependent manner (r = 0.120, *p* = 0.023), and a similar association was observed for energy drinks. Our findings reaffirmed that caffeine is an evident risk factor for insomnia. Caffeine acts by antagonizing adenosine receptors, leading to increased alertness and delayed sleep onset [3]. Increased coffee consumption is associated with sleep fragmentation and reduced total sleep duration. Among students, during intellectual overload academic periods, coffee consumption increases, which can lead to sleep deprivation and additional stimulant use to maintain cognitive performance, thus creating a vicious cycle [21,36]. Similar to our results, energy drinks have been correlated in other studies with an increased rate of insomnia, the cumulative effect on the central nervous system generating hyperexcitability, difficulty maintaining sleep, and circadian rhythm disturbances during the examination period [3,33].

Merwid-Ląd et al. [34] reported that insomnia and sleep disorders were identified in approximately 40% of health science students, ranking among the most common adverse effects. Thus, sleep disorders represent some of the most common adverse reactions reported by users of cognitive enhancers, with a bidirectional effect: the use of CEs causes sleep disorders, and sleep deprivation leads to increased compensatory consumption of CEs [3,33]. All of these can lead to the progressive worsening of chronic fatigue, a decline in long-term cognitive performance, and impaired overall health, creating an imbalance between benefits and risks.

Regarding other adverse effects associated with CE use, our study identified statistically significant increases in tachycardia and palpitations with caffeine consumption. In fact, caffeine intake is the only significant independent predictor of cardiovascular events identified. Similarly described in other studies, tachycardia is one of the most frequently reported adverse reactions, being particularly associated with the consumption of caffeine and other stimulants [34]. When using pharmacological cognitive enhancers, other cardiovascular effects may also occur, such as increased blood pressure and palpitations, particularly under conditions of uncontrolled use [3]. In a study assessing the prevalence and perceived benefits of caffeinated beverage consumption among university students, over 98.5% of participants consumed caffeine, 31% reported being caffeine-dependent, and excessive caffeine consumption was significantly associated with cardiac problems [50].

Among the students analyzed in our study, anxiety triggers a stimulation-seeking behavior that exacerbates sleep deprivation. Numerous studies similarly highlight the emergence of neuropsychiatric symptoms, such as anxiety, irritability, agitation, and mood disorders [3]. Prolonged use or overdose can lead to episodes of severe anxiety or paradoxical cognitive impairment [47].

Our data indicated that self-medication practices significantly increase the risk of adverse outcomes by 3.05 times, whereas prescription-based procurement multiplies this likelihood by 5.12 times. In accordance with our findings, the literature describes tolerance and dependence, particularly with the use of high doses of pharmacologically active stimulants [3,51]. Users may also experience headache, tremor, gastrointestinal disturbances, decreased appetite, and nervousness, especially with combined use [3].

*Cognitive enhancers and academic anxiety: the contrasting effect of academic advancement*

Regarding amphetamine and psychoactive substance use, the results of the study highlighted that higher academic anxiety predicts substance use. For these substances, academic advancement decreases the use of them, playing a role as a protective factor.

Regarding the use of synthetic nootropics, such as piracetam and cerebrolysin, we found that the only significant independent predictor was the year of study (OR = 1.86, *p* = 0.021. We can explain this by several possible causes: as the student progresses in the years of study, the workload and information increase as studies move from preclinical to clinical years; the stress adaptation mechanism is no longer effective; knowledge in the medical and pharmacological fields makes them give in more easily and choose a shorter path in the face of academic challenges; and the influence of the group of colleagues who use this substance makes it become a normal, habitual, accepted behavior.

So, anxiety serves as a catalyst for the use of amphetamines and psychoactive substances. Interestingly, advancing in years of study has a contrasting effect: the use of illicit substances decreases, and the use of synthetic nootropics increases. This highlights a strategy that medical students adopt during the academic years: knowledge about pharmaceutical substances, the effect of the use of substances on mental and physical health, the risks when comparing the two categories, and the possibility of developing addiction. It is very likely that information about the effects of the studied substances leads them to choose nootropics for cognitive stimulation and lack of addiction over amphetamines and psychoactive substances that make them feel energetic for a short period of time but cause addiction.

*Cognitive enhancers use and academic dishonesty*

From an ethical point of view, this consumption is worrying. Students in medical specialties know or can easily find out for what purpose these drugs are used and the effects (e.g., piracetam and cerebrolysin are used in neurological diseases). Their consumption by medical science students shows that there is a tendency to minimize the risks of consumption to maximize academic results.

Therefore, intervention methods in medical universities should aim not only at prevention but also at psychological intervention to help students deal with academic stress through appropriate coping methods that are less risky for their health.

An important finding in our study is that most students do not consider the use of cognitive enhancers to be academic dishonesty. However, the literature addresses issues related to the authenticity of academic performance, with debates focusing on whether the results obtained in this way truly reflect the student’s individual abilities or are the result of a pharmacological “boost” [52]. The literature also addresses issues of social justice and access, suggesting that the use of cognitive enhancers may exacerbate inequalities and that individuals with easier access to these substances may gain competitive advantages, raising questions regarding fairness in the educational environment (from neurotherapeutics to ethical and regulatory challenges). In contexts such as sports or academic performance, use may be associated with “cognitive doping,” which calls into question individual merit and effort [53,54,55,56,57].

Cognitive stimulant use must be addressed as an emerging public health issue, not just academic behavior, requiring the development of university guidelines and preventive screening methods. From a public health perspective, there is a need for further research to quantify exposure levels, conduct biomonitoring studies, integrate clinical and toxicological assessments, and assess long-term health consequences.

The use of CEs should concern both students and academic staff. On the one hand, the use of CEs falls within a category of unethical behavior that is encouraged by the lack of institutional intervention but also by the student community. The behavior becomes normal if it is practiced by many colleagues and is not sanctioned by the institution. Secondly, it raises problems regarding the professional path of the student, a future doctor, who resorts to such mechanisms to improve his cognitive performance. Is breaking the rules during his student life associated with breaking the rules in his professional career? There are some questions that may concern professionalism, decision-making situations, and integrity.

### 4.1. Strengths and Limitations of the Study

The results of the present study are important first because they complete the information on substance use among healthcare students in Romania, as there is not much conclusive data published previously. Second, the study identifies the level of academic anxiety and the role of advancing in the year of study as protective factors regarding the use of illicit substances. The results also add information about the increase in the use of cognitive stimulants and highlight the role of academic anxiety.

Among the limitations of the study, we mention the lack of gender balance, so that the results cannot be generalized when comparing students according to gender. Additionally, the performance of a large number of simultaneous statistical analyses may inflate the Type I error rate. Therefore, marginal associations should be interpreted with caution. Also, this research used a non-probability convenience sampling method by distributing the questionnaire online to available students, so we cannot generalize the results for other populations of non-medical students [58].

### 4.2. Reflections and Planning

The results highlight a significant difference between freshmen and senior students. Therefore, universities should consider the high risk of burnout and mental health problems among students. Academic evaluation methods as well as psychological interventions must be differentiated depending on the year of study. Academic staff and administrators should find ways to reduce anxiety and stress among first-year students by dividing activities or assessments and offering psychological support. For senior students, academic staff should focus on prevention, self-medication, and strategies for managing stress and burnout.

## 5. Conclusions

The use of cognitive enhancers among students is largely motivated by academic factors and the desire to achieve performance. Factors associated in the literature with risky behaviors, such as sex or sleep deprivation, did not demonstrate predictive value in our research, indicating that anxiety takes precedence over isolated demographic or biological factors. Advancing in years of study becomes a protective factor when it comes to the use of addictive illicit substances. Medical students do not perceive the use of cognitive enhancers as academic fraud, but it raises complex ethical issues related to fairness, social pressure, and the authenticity of performance.

## Figures and Tables

**Table 1:** Socio-demographic, anthropometric, and lifestyle characteristics (N = 402) ^1^.

Characteristics	M ± SD and N (%)
Gender	
Male	85 (21.1%)
Female	317 (78.9%)
Environment	
Urban	273 (67.9%)
Rural	129 (32.1%)
Marital status	
Single	194 (48.3%)
In a relationship	208 (51.7%)
Life-living data	
Single	80 (19.9%)
With family	127 (31.6%)
Shared housing (dormitory, rental)	195 (48.5%)
Professional status	
Unemployed	351 (87.3%)
Employed	51 (12.7%)
Body mass index (BMI)	
Male	24.88 ± 4.59
Female	22.54 ± 3.84
Weight status (WHO)	
Underweight	34 (8.5%)
Normal	262 (65.5%)
Overweight	81 (20.3%)
Obesity grades 1, 2, and 3 (cumulative)	23 (5.8%)
Physical health (self-assessment 1–5)	
1 (very bad)/2 (bad)	24 (6.0%)
3 (average)	134 (33.3%)
4 (good)/5 (excellent)	244 (60.7%)
I suffer from a chronic disease	
No	345 (85.8%)
Yes	57 (14.2%)
Smoking behavior	
Yes	103 (25.6%)
On average, I sleep per night	
Under 6 h	120 (29.9%)
Over 6 h	282 (70.1%)

^1^ Means and standard deviations (M ± SD), absolute frequencies (n) and percentages (%). Percentages reflect the number of valid responses for each variable.

**Table 2:** Frequency of consumption of common substances at the sample level (N= 402) ^1^.

Substance Consumed	Never	Rarely	Frequently	Daily
Coffee	45 (11.2%)	78 (19.4%)	69 (17.2%)	210 (52.2%)
Carbonated drinks	30 (7.5%)	234 (58.2%)	119 (29.6%)	19 (4.7%)
Alcohol	86 (21.4%)	284 (70.6%)	30 (7.5%)	2 (0.5%)
Energy drinks	267 (66.4%)	104 (25.9%)	26 (6.5%)	5 (1.2%)
Drugs (recreational)	396 (98.5%)	5 (1.2%)	0(0.0%)	1 (0.2%)

^1^ Absolute frequency (n) and percentage (%).

**Table 3:** Chi-square test results for variables associated with stimulant-induced insomnia (n = 363) ^1^.

Associated Variable	χ^2^	*df*	*p*
Sleep Duration (<6 h vs. >6 h)	5.250	1	0.022
Energy Drink Consumption Frequency	19.399	3	<0.001
Piracetam/Cerebrolysin Use	13.101	2	0.001

^1^ χ^2^ = Pearson chi-square statistic; *df* = degrees of freedom. *p* = statistical significance level.

**Table 4:** The use, efficacy, and ethical perception of cognitive stimulants ^1^.

Parameters Analyzed	N (%)
Frequency of administration (N = 362)	
A few times a year	148 (40.9%)
A few times a month	57 (15.7%)
A few times a week	84 (23.2%)
Daily	73 (20.2%)
How to purchase without a prescription (N = 386)	
Yes	258 (66.8%)
No	128 (33.2%)
Main source of purchase (N = 402)	
Pharmacy	326 (81.1%)
Online environment	48 (11.9%)
Awareness of the risk of addiction (N = 402)	
No concerns	331 (82.3%)
Concerned (Yes/Maybe)	71 (17.7%)
Do you consider using a form of cheating? (N = 402)	
No	382 (95.0%)
Yes	20 (5.0%)
Should they be banned in universities? (N = 395)	
No	351 (88.9%)
Yes/Maybe	44 (11.1%)

^1^ Absolute frequency (n) and percentage (%).

**Table 5:** Logistic regression coefficient for predicting the occurrence of adverse effects ^1^.

Predictors	B	SE B	Wald	*p*	OR	95% CI for OR
Medical prescription	1.634	0.511	10.213	0.001	5.125	1.881–13.964
Self-medication	1.116	0.530	4.437	0.035	3.053	1.081–8.626
Concern about dependence	0.831	0.251	10.949	0.001	2.296	1.403–3.758
Academic anxiety level	−0.002	0.026	0.005	0.941	0.998	0.949–1.049

^1^ B = unstandardized regression coefficient; SE B = standard error of the coefficient; Wald = Wald test statistic; *p* = statistical significance level; OR = odds ratio. The assumed significance level was *p* < 0.05. Model fit indicators: Omnibus χ^2^ (4) = 23.402, *p* < 0.001; Nagelkerke R^2^ = 0.147.

**Table 6:** Coefficients of multiple logistic regression models for predicting cognitive benefits ^1^.

Dependent Variable	Predictors	B	SE B	Wald	*p*	OR	95% CI for OR
Model 1: Perceived Attention/Vigilance	*Ginkgo biloba*	0.846	0.261	10.473	0.001	2.330	1.396–3.888
	*Lecithin*	0.807	0.264	9.333	0.002	2.241	1.335–3.759
Model 2: Perceived Short-Term Memory	*Ginkgo biloba*	0.661	0.237	7.802	0.005	1.938	1.218–3.082
	*Lecithin*	0.368	0.237	2.408	0.121	1.444	0.908–2.298
Model 3: Perceived Long-Term Memory	*Ginkgo biloba*	0.282	0.225	1.560	0.212	1.325	0.852–2.062
	*Lecithin*	0.839	0.226	13.802	<0.001	2.313	1.486–3.600
Model 4: Perceived Academic Performance	*Ginkgo biloba*	0.825	0.240	11.840	0.001	2.282	1.426–3.652
	*Lecithin*	0.729	0.241	9.171	0.002	2.073	1.293–3.322

^1^ B = unstandardized regression coefficient; SE B = standard error; Wald = test statistic; *p* = statistical significance level; OR = odds ratio. All four regression models were highly statistically significant (Omnibus χ^2^ tests, *p* < 0.005). Terminology adjusted to reflect subjective perception.

**Table 7:** Parameters of logistic regression models: independent predictors for adverse and addictive reactions ^1^.

Evaluated Effect (Dependent Variable)	Predictor	B	SE B	Wald	*p*	OR	95% CI for OR
Cardiovascular Disorders	*Caffeine*	1.084	0.330	10.792	0.001	2.958	1.549–5.649
	*Theanine*	0.854	0.656	1.698	0.193	2.350	0.650–8.496
Insomnia	*Caffeine*	0.672	0.276	5.944	0.015	1.958	1.141–3.361
	*Theanine*	0.553	0.645	0.735	0.391	1.739	0.491–6.158
Tolerance	*Caffeine*	0.390	0.289	1.812	0.178	1.476	0.837–2.603
	*Theanine*	1.602	0.623	6.612	0.010	4.965	1.464–16.843
Dependence	*Caffeine*	1.565	0.639	6.006	0.014	4.782	1.368–16.716
	*Theanine*	2.593	0.678	14.633	<0.001	13.367	3.541–50.461

^1^ B = unstandardized regression coefficient; SE B = standard error; Wald = test statistic; OR = odds ratio; *p* = statistical significance level. Significance level set at *p* < 0.05.

**Table 8:** Multiple logistic regression model parameters for predicting the use of amphetamines or other psychoactive substances (N = 336) ^1^.

Predictor Variables	B	S.E.	Wald	*p*	OR	95% CI for OR
Academic anxiety score	0.145	0.054	7.188	0.007	1.16	1.040, 1.285
Year of study (Advancement)	−0.872	0.348	6.275	0.012	0.42	0.211, 0.827
Gender	−1.281	0.809	2.508	0.113	0.28	0.057, 1.356
Average hours of sleep	−0.892	0.818	1.189	0.276	0.41	0.082, 2.037

^1^ B = unstandardized regression coefficient; SE B = standard error; Wald = test statistic; OR = odds ratio; *p* = statistical significance level. Significance level set at *p* < 0.05. Model Omnibus χ^2^ (4) = 22.398, *p* < 0.001; Nagelkerke R^2^ = 0.320.

## Data Availability

The raw data supporting the conclusions of this article will be made available by the authors on request.

## Abbreviations

The following abbreviations are used in this manuscript:

M	mean
SD	standard deviation
N	number of participants
Mdn	median
